# Typho-Malarial Fever

**Published:** 1887-09

**Authors:** 


### TYPHO-MALARIAL FEVER.

This form of fever is more than usually prevalent the present season, and is very obstinate, resisting quinine and all other agents given to cut it short. It seldom terminates under two septinary periods, or two weeks, and frequently runs to four, five or six weeks. In most cases during the first two weeks the stomach is irritable, the tongue red and pointed at the tip, and the patient lothes food in any and every shape.

The common and stereotyped instruction in books and journals to nourish your patient from the very start *is a humbug*, which we old and experienced doctors well understand. The patient can't eat, or won't eat, simply because, in most cases, his stomach rejects it; and if his stomach does not reject it there is no digestion, no appropriation of food, and the nourishment given under these circumstances is worse than useless, distressing the patient, increasing the fever, and often running off at the bowels and causing a troublesome diarrhœa.

The patient never wants food until the fever begins to abate, and then it should be allowed in milk and broths. Butter milk, a half glass at a time, will often be found best, especially if there is thirst and the patient craves acids. Later, milk toast, rice, milk and soft boiled eggs may come in, and then a little broiled beef, or chicken, with coffee or tea, if the patient has been accustomed to these drinks.

The expectant plan of treatment is by far the best and safest.

Quinine is of little use, and, indeed, is often injurious. It should be given only when the temperature is at 100 or less, and then only at the septenary periods when nature seeks to terminate the fever. The use of very large doses as antipyretic, we think, has done much harm. When the temperature rises above 102½, antifebrine in 5-grain doses, once or twice a day, will be found far more efficient than quinine to lower the temperature. Often sponging the skin with quinine and whisky will suffice to bring down the temperature.

Apart from this the physician can only watch the case through its course, meeting symptoms as they arise and guarding against local inflammation.

